# Hidradenitis suppurativa in primary care: From early recognition to modern management

**DOI:** 10.4102/safp.v68i1.6318

**Published:** 2026-09-04

**Authors:** Ilana Steenkamp, Leantha Pillay

**Affiliations:** 1 Department of Dermatology, Robert Mangaliso Sobukwe Hospital, Kimberley, South Africa; 2 Department of Dermatology, Faculty of Health Sciences, University of the Free State, Bloemfontein, South Africa

**Keywords:** Hidradenitis suppurativa, primary care, diagnosis, management, recognition, family practitioners

## Abstract

Hidradenitis suppurativa (HS), also known as acne inversa, is a heterogeneous, chronic, relapsing inflammatory disease of the pilo-sebaceous unit, primarily affecting intertriginous areas such as the axillae, groin and anogenital region. Despite its relatively low global prevalence, HS remains underdiagnosed, especially in primary care settings, where it is often mistaken for recurrent boils. The pathogenesis is multifactorial, involving follicular occlusion, immune dysregulation, genetic susceptibility, microbiome imbalance, hormonal influences and lifestyle factors. Hidradenitis suppurativa typically presents after puberty with a peak incidence during the second and third decades of life. The condition is characterised by painful nodules, abscesses and in advanced stages of the disease, forms sinus tracts and scars that greatly impair quality of life. Early recognition and a multidisciplinary management approach, including lifestyle modification, long-term medical therapy and surgical intervention when indicated, are essential to prevent disease progression and complications. This article provides an evidence-based overview of HS and practical guidance for family practitioners on its recognition, classification and management within the South African context.

## Introduction

Hidradenitis suppurativa (HS) is a chronic, relapsing inflammatory skin disorder affecting intertriginous areas such as the axillae, groin, perineum and inframammary regions. It typically presents in young adults with painful, deep-seated nodules and abscesses that may resolve or persist, often leading to sinus tracts and scarring.^1,2,3^ Despite its physical and psychological burden, HS is under-recognised, particularly in primary care. In South Africa, HS occurs across all ethnic and socioeconomic groups. Limited awareness, underreporting, misdiagnosis and cultural perceptions of skin disease contribute to an underestimated prevalence.^2^ Diagnosis is challenging, as HS is often mistaken for recurrent furunculosis or abscesses, delaying accurate identification by years and worsening morbidity and quality of life.^4,5^ Its unpredictable, relapsing course further complicates management, causing frustration for patients and clinicians alike. Optimal care requires long-term, multimodal therapy, including lifestyle changes, topical and systemic treatments, surgical intervention and psychosocial support.^3,4^ This article provides an overview of HS for South African family physicians, emphasising key clinical features, diagnostic challenges, and the importance of early, holistic management to improve patient outcomes.

## Pathogenesis

The pathophysiology of HS is multifactorial and incompletely understood, involving follicular occlusion, immune dysregulation, genetic susceptibility, hormonal influences and environmental factors^1^ (Figure 1).

**FIGURE 1:**
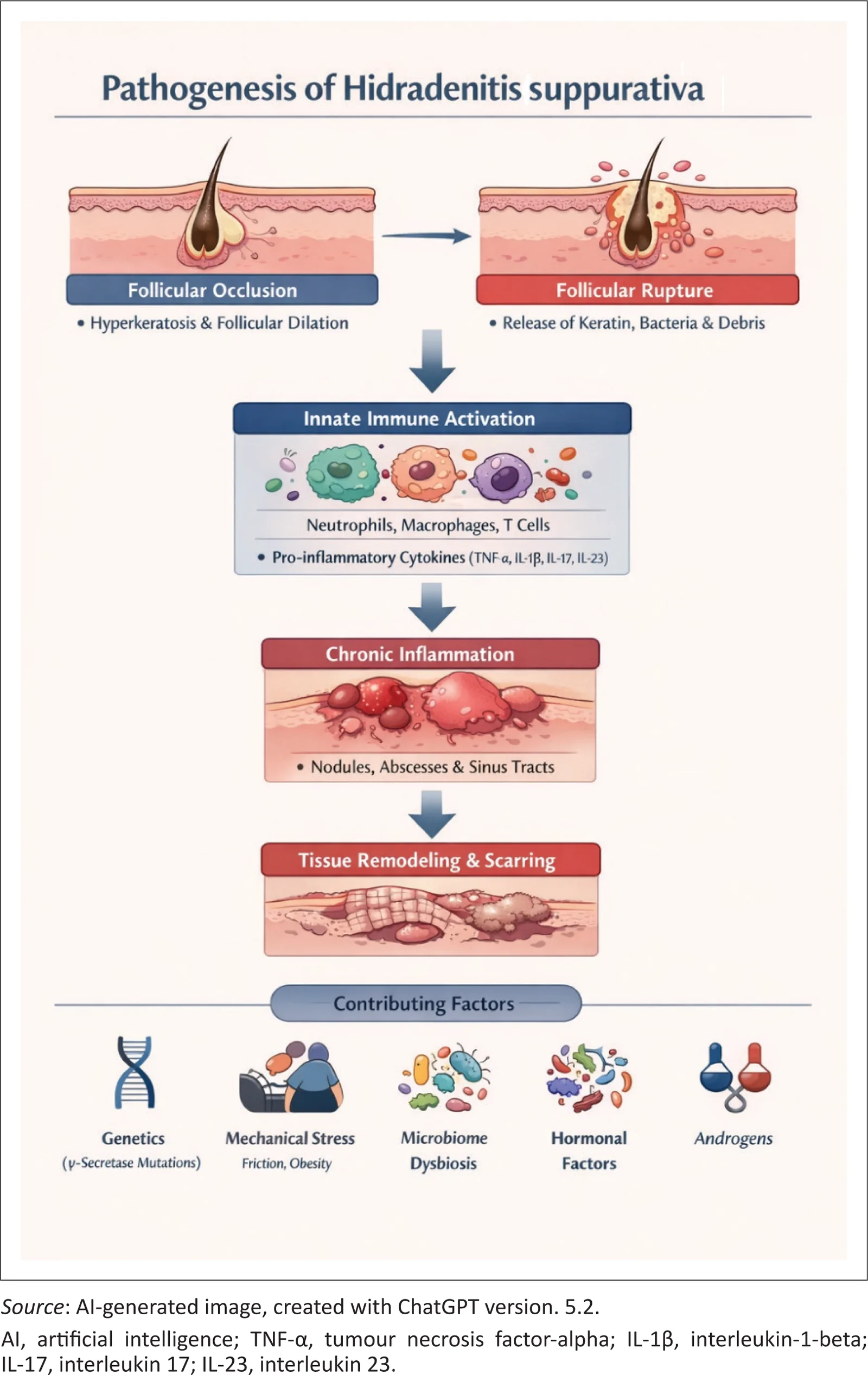
Pathogenesis of Hidradenitis suppurativa.

### Follicular occlusion and rupture

The primary event in HS is follicular occlusion within the pilosebaceous unit.^6^ Abnormal keratinisation leads to follicular plugging and dilation, followed by rupture and release of keratin, bacteria and debris into the dermis, triggering a marked inflammatory response.^7,8^

### Immune dysregulation

Follicular rupture activates both innate and adaptive immune pathways, resulting in increased pro-inflammatory cytokines, including tumour necrosis factor alpha (TNF-α), interleukin beta (IL-1β), IL-17 and IL-23. These cytokines sustain chronic inflammation, tissue damage and scarring.^4,7,8^

### Microbial factors

Hidradenitis suppurativa is not primarily infectious; however, secondary bacterial colonisation of ruptured follicles and sinus tracts may perpetuate inflammation. Biofilm formation in chronic lesions contributes to disease persistence and treatment resistance.^6,7,8^

### Genetic and hormonal influences

Approximately one-third of patients report a family history, indicating a genetic component.^9^ Mutations in γ-secretase complex genes involved in follicular keratinisation and Notch signalling have been identified in some familial cases.^4,10^ Hormonal factors are implicated, as HS typically begins after puberty, may flare during the premenstrual period, and often improves after menopause.^3,8^

### Environmental and lifestyle factors

Smoking and obesity are strongly associated with HS. Nicotine promotes follicular plugging and inflammation, while obesity increases mechanical friction, hormonal imbalance and systemic inflammation.^3,8,9^

## Risk factors

Understanding the risk factors for HS is essential for early recognition, prevention and optimal patient counselling.^1,9^ Risk factors are broadly categorised as non-modifiable (inherent) and modifiable or environmental.^3,4,7^ Their interaction assists clinicians in identifying at-risk individuals and implementing timely interventions to reduce disease burden (Table 1).

**TABLE 1:** Risk factors in Hidradenitis suppurativa.

Category	Risk factor	Clinical relevance
Non-modifiable	Age	Onset typically in late adolescence to early adulthood; peak incidence in late teens-early 20s, coinciding with apocrine gland activity and hormonal changes.
	Gender	More common in women; likely reflects hormonal influences, immune differences, fat distribution, and healthcare-seeking behaviour.
	Family history and genetics	20-40% report affected relatives; y-secretase complex mutations identified in some familial cases but explain a minority of HS overall.
	Ethnicity	Higher prevalence and severity in individuals of African ancestry, likely due to combined genetic, environmental, and healthcare access factors.
Modifiable and/or Environmental	Metabolic syndrome	Obesity, high BMI, type 2 diabetes, and cardiovascular disease increase HS risk and severity; weight loss improves disease control.
	Smoking	Strongly associated with HS severity; nicotine promotes follicular occlusion, chronic inflammation, oxidative stress, endothelial dysfunction, and microbiome alteration.
	Mechanical stress	Friction, occlusion, and microtrauma in intertriginous areas promote follicular rupture; exacerbated by tight clothing, prolonged sitting, repetitive movement, and obesity.
	Hormonal influences	Post-pubertal onset, perimenstrual flares, PCOS association, and response to anti-androgens suggest hormones modulate disease expression rather than act as primary causes.
	Delayed diagnosis	Diagnostic delay (7-10 years in adults; 1-2 years in children) leads to scarring, sinus tract formation, advanced disease, poorer treatment response, and increased comorbidities.

*Source:* AI-generated table created using ChatGPT version. 5.2.

AI, artificial intelligence; BMI, body mass index; HS, Hidradenitis suppurativa; PCOS, polycystic ovary syndrome.

## Clinical features

Hidradenitis suppurative typically affects intertriginous areas such as the axillae, groin, perineum and inframammary folds, with less frequent involvement of the face, neck, post-auricular region and trunk.^1,3,7^ Early signs include open and closed comedones, particularly ‘double-ended’ or ‘bridging’ comedones, along with inflammatory papules, tender nodules and abscesses. Chronic disease can lead to sinus tracts, fistulas and dense, rope-like scars (Figure 2).^3,4^ Draining sinuses often produce malodorous serosanguinous or purulent discharge, worsening psychosocial burden and quality of life. Hidradenitis suppurativa is relapsing and progressive, predisposing to secondary infections, lymphedema, severe scarring causing urethral, anal or rectal strictures and limb contractures. Rarely, chronic lesions may develop into squamous cell carcinoma.^3,7^ The disease is profoundly debilitating, strongly associated with depression, anxiety, sexual dysfunction and social withdrawal, significantly affecting patients and their families.^1,8,11^

**FIGURE 2:**
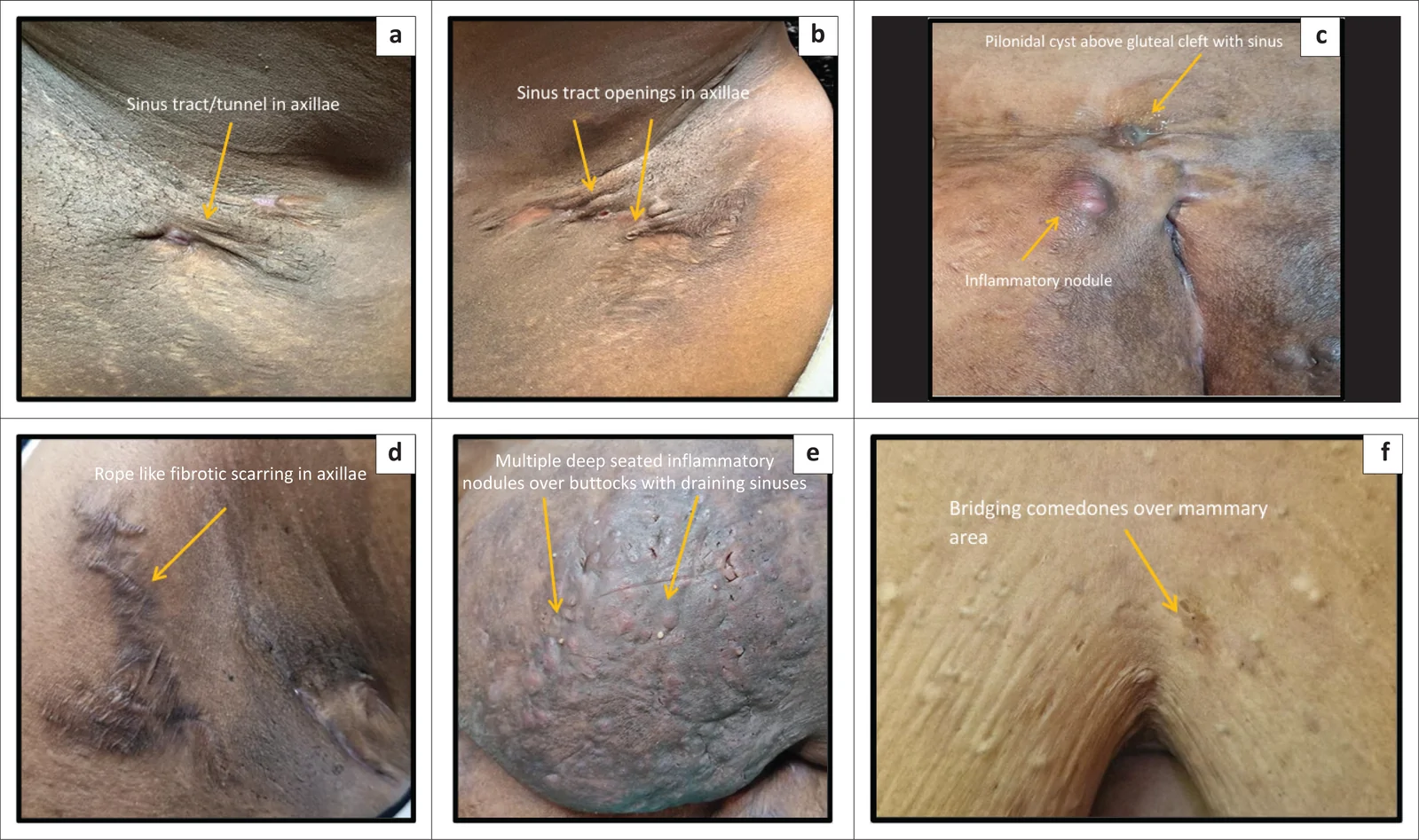
Skin changes in Hidradenitis suppurativa.

## Concomitant diseases

Hidradenitis suppurativa is increasingly recognised as a systemic inflammatory disorder with multisystem comorbidities. Metabolic and cardiovascular diseases are common; metabolic syndrome affects up to 50% of patients.^11,12^ increasing the risk of type 2 diabetes, atherosclerotic disease, myocardial infarction and stroke, with a 70% higher stroke risk.^8,13,14^ Annual screening of waist circumference, blood pressure, lipids and glucose is recommended.^11^

Non-alcoholic fatty liver disease occurs in over half of patients, independent of obesity, reflecting shared inflammatory pathways and risk of progressive liver disease. Hidradenitis suppurativa also carries a significant psychosocial and psychosexual burden: sexual dysfunction affects up to 60% of patients, depression up to 30% of adults (16% of children)^5,15^ and anxiety up to 20%, roughly double the population rates.^8,11,16^ Inflammatory and endocrine comorbidities are overrepresented. Women have higher odds of polycystic ovarian syndrome, while inflammatory arthritis and inflammatory bowel disease, particularly Crohn’s, are more frequent than in the general population.^7,10,11^

Hidradenitis suppurativa is linked to reduced life expectancy due to cardiovascular events, stroke and malignancy.^4,11^ Early recognition and proactive screening are essential to reduce long-term morbidity and mortality.

## Autoinflammatory syndromes associated with Hidradenitis suppurativa

Hidradenitis suppurativa may rarely occur within autoinflammatory syndromes driven by neutrophilic inflammation and multisystem involvement. These include PASH (pyoderma gangrenosum, acne, HS), PAPASH (with sterile pyogenic arthritis), PsAPASH (with psoriatic arthritis) and PASS (associated with ankylosing spondylitis, with variable HS expression).^10,14^ Syndromic HS is typically more severe, with prominent systemic inflammation and musculoskeletal or neutrophilic features, often requiring multidisciplinary management and targeted immunomodulatory therapy such as TNF-α or IL-1 inhibitors.^4,10,14^ Although exceptionally rare, recognising syndromic HS is important due to its severity and need for specialist care.

## Severity assessment tools in Hidradenitis suppurativa

Accurate severity assessment in HS guides management and monitoring. Despite more than 30 proposed tools, no gold standard exists,^10^ and most classify severity rather than treatment response. The most practical tools for the clinician are the refined Hurley classification, HS–Physician’s Global Assessment (HS-PGA) and the International HS Severity Scoring System (IHS4).^10^

## Refined Hurley staging system

This system evaluates sinus tracts, inflammation and the number of affected anatomical regions, creating seven substages^1^.

Stage I: Abscesses without sinus tracts or scarring

- IA: Single lesion in one area
- IB: Multiple lesions in one area

Stage II: Recurrent abscesses with sinus tracts and scarring, separated by normal skin

- IIA: Mild involvement (few tracts or abscesses)
- IIB: Moderate involvement (multiple tracts and abscesses in one area)

Stage III: Diffuse or near-diffuse disease with multiple interconnected tracts and abscesses

- IIIA: Diffuse involvement in one region
- IIIB: Extensive involvement in multiple regions

## Patient outcome severity tools

Patient outcomes in HS are measured using validated tools assessing pain, symptoms and quality of life. Pain is scored on a 10-point visual analogue scale, while disease impact, symptom burden and quality of life are evaluated using the HS Impact Assessment (HSIA), HS Symptom Assessment (HSSA) and the HS Quality of Life scale (HiSQOL), respectively, providing a comprehensive assessment of patient experience.^1,4,10^

## Management

No South African–specific guidelines exist for the management of HS. Clinical practice may therefore be informed by international guidance, including the 2024 European guidelines, which recommend a severity-based, stepwise approach incorporating general measures, antibiotics, anti-inflammatory therapies, hormonal treatment, biologics, retinoids, pain management and surgical therapy (Table 2).^11,12,17^

**TABLE 2:** Management of Hidradenitis suppurativa.

Management summary of Hidradenitis suppurativa			
Therapy category	Indications	Treatment options	Key notes
Topical agents	Mild HS (Hurley I); adjunctive	• Clindamycin 1% BD (8-12 weeks and maintenance); • Resorcinol 15%; antiseptic washes	Anti-inflammatory; Monitor for contact dermattis
Intralesional therapy	Acute painful nodules	• Triamcinolone 5-10 mg/mL	Rapid pain and inflammation relief
Wound care	Draining and/or open lesions	• Silicone foam; • Hydrofiber; • Silver dressings; • Short-term NPWT	Individualise by exudate, site, tolerance
Systemic antibiotics (1st line)	Moderate HS (Hurley II)	• Doxycycline 100 mg BD; • Minocycline 100 mg daily (8-12 weeks)	Primarily anti-inflammatory
Systemic antibiotics (2nd line)	Refractory and/or extensive disease	• Clindamycin 300 mg BD + Rifampicin • 300 mg BD (10-12 weeks)	Monitor LFTs and drug interactions
Severe antibiotic regimens	Severe, recalcitrant HS	• Metronidazole + Moxifloxacin + • Rifampicin (6-12 weeks) IV Ertapenem (6 weeks)	Ertapenem often bridge to surgery
Hormonal therapy	Perimenstrual flares, PCOS, hyperandrogenism	• COCP; *Spironolactone*; • Finasteride; Metformin	Particularly effective in women
Systemic retinoids	Selected refractory disease	• *Isotretinoin*; Acitretin	Highly teratogenic
Systemic corticosteroids	Severe acute flares	• Short tapering courses	Rescue therapy only
Biologic therapy	Moderate-severe refractory HS	• Adalimumab; Secukinumab; Bimekizumab; • Infliximab	Screen for TB, HIV, hepatitis
Laser therapies	Adjunct or recurrent disease	• Nd:YAG; CO_2_; Hair reduction lasers	Reduce inflammation and recurrence

*Source:* AI-generated table created using ChatGPT version. 5.2.

AI, artificial intelligence; CO_2_, carbon dioxide; COCP, combined oral contraceptive pill; I&D, Incision & Drainage, LFT, liver function test; Nd:YAG, neodymium doped yttrium aluminium garnet; NPWT, negative pressure wound therapy; PCOS, polycystic ovary syndrome.

### General measures

General care underpins HS management and includes patient education, modification of risk factors, pain control, wound care and psychosocial support.^11,17^ These measures should accompany all medical and surgical interventions.

### Topical therapy

Topical agents are suitable for mild disease or as adjuncts to systemic therapy:

- Non-antibiotics: Resorcinol 15% (keratolytic and antiseptic) applied twice daily during flares and once daily as maintenance for up to 16 weeks.^12,17^
- Antibiotics: Clindamycin 1% applied twice daily during flares for up to 12 weeks.^12,17^

### Systemic antibiotics

These are mainly used for their anti-inflammatory effects in moderate-to-evere HS.

- Tetracyclines: First-line systemic therapy for 3 months.^12,17^
- Clindamycin plus rifampicin: 300 mg twice daily each for 10–12 weeks in selected patients.^12,17^
- Clindamycin monotherapy: 300 mg twice daily orally for 12 weeks, or 600 mg three times daily intravenously for 5 days when combination therapy is unsuitable.^12,17^
- Ertapenem: 1 g intravenously daily for 6 weeks in severe or refractory disease or as preoperative down-staging.^12,17^
- Alternative regimens: Clindamycin–ofloxacin or rifampicin–moxifloxacin–metronidazole combinations (limited evidence).^12,17^

Prolonged antibiotic use should be carefully weighed against the risk of antimicrobial resistance, with specialist input in complex cases.

### Anti-inflammatory and immunomodulatory therapy

Patients with ongoing inflammatory disease despite antibiotics may require additional anti-inflammatory or immunomodulatory agents, often alongside surgical management. Options include intralesional or systemic corticosteroids, dapsone, colchicine, ciclosporin, zinc gluconate and immunoglobulins.^12,17^

### Hormonal therapy

Hormonal treatment may benefit selected women, particularly those with perimenstrual flares or polycystic ovary syndrome, although supporting evidence is limited.^12,17^

### Biologic therapy

Biologic agents targeting key inflammatory pathways (e.g., TNF-α, IL-17) are indicated for moderate-to-severe HS unresponsive to conventional therapy and should be initiated and monitored in specialist care settings. Adalimumab, a TNF-α inhibitor, has the strongest evidence base for the treatment of moderate-to-severe HS, supported by phase III randomised controlled trials; secukinumab is supported by emerging phase III trial evidence; and infliximab has moderate quality evidence from smaller trials and observational studies supporting off-label use in selected patients.^12,17,18,19^

### Retinoids

Retinoids have variable efficacy in HS. Isotretinoin may be considered at a dose of 0.5 mg/kg/day – 1.2 mg/kg/day for 4–12 months, while acitretin may be useful in early disease.^12,17^

### Pain management

Pain is often severe, requiring a multimodal approach. Acute flares are managed with non-steroidal anti-inflammatory drugs (NSAIDs), such as acetaminophen, or short courses of opioids for severe pain. Adjunctive therapies include topical anaesthetics, intralesional corticosteroids and neuropathic pain agents.^12,17^

### Surgical management

Surgical intervention is indicated for refractory or advanced disease, including incision and drainage of abscesses, limited excision and wide local excision with primary closure or grafting. Reconstructive techniques may reduce recurrence and improve function. Surgery is often combined with medical therapy to optimise outcomes, prevent recurrence and improve quality of life.^11,17^

## Conclusion

Hidradenitis suppurativa is a chronic relapsing disease with profound physical, psychological and social consequences. Early recognition, careful assessment of disease severity, and a personalised, stepwise management strategy are essential for optimal care. Integrating lifestyle interventions, topical and systemic therapies, biologics and surgical or procedural treatments improves outcomes and quality of life. Equally important is addressing comorbidities, providing patient education and offering psychosocial support. Recent advances in biologic therapies have transformed management for moderate-to-severe disease, yet continued research, especially in the South African setting, is needed to refine treatment approaches, standardise outcomes, and further improve long-term patient care.
